# The Role of Trace Elements in Cardiovascular Diseases

**DOI:** 10.3390/toxics11120956

**Published:** 2023-11-23

**Authors:** Christian Wechselberger, Barbara Messner, David Bernhard

**Affiliations:** 1 Division of Pathophysiology, Institute of Physiology and Pathophysiology, Medical Faculty, Johannes Kepler University Linz, 4020 Linz, Austria; 2 Cardiac Surgery Research Laboratory, Department of Cardiac Surgery, Medical University of Vienna, 1090 Vienna, Austria; barbara.messner@meduniwien.ac.at; 3 Clinical Research Institute for Cardiovascular and Metabolic Diseases, Medical Faculty, Johannes Kepler University Linz, 4020 Linz, Austria

**Keywords:** cadmium, trace elements, cardiovascular disease

## Abstract

Essential trace elements play an important role in human physiology and are associated with various functions regulating cellular metabolism. Non-essential trace elements, on the other hand, often have well-documented toxicities that are dangerous for the initiation and development of diseases due to their widespread occurrence in the environment and their accumulation in living organisms. Non-essential trace elements are therefore regarded as serious environmental hazards that are harmful to health even in low concentrations. Many representatives of these elements are present as pollutants in our environment, and many people may be exposed to significant amounts of these substances over the course of their lives. Among the most common non-essential trace elements are heavy metals, which are also associated with acute poisoning in humans. When these elements accumulate in the body over years of chronic exposure, they often cause severe health damage in a variety of tissues and organs. In this review article, the role of selected essential and non-essential trace elements and their role in the development of exemplary pathophysiological processes in the cardiovascular system will be examined in more detail.

## 1. Introduction

Heavy metal trace elements are found as natural components of the Earth’s crust. However, especially since the age of the industrial revolution starting in the mid-18th century, anthropogenic activities have created a major problem with regard to the aspects of environmental pollution and the poisoning of food chains [1,2]. In particular, the accumulation of heavy metals that enter the organism as part of people’s daily activities, e.g., through food, cosmetics or the abovementioned increasing pollution of the environment, represents an ever-increasing problem [3,4,5].

Inorganic elements that are present in low concentrations in body tissues or fluids are colloquially called trace elements. For several of these trace elements, at least one important physiological function in human metabolism is described [6]. Deficiency in essential trace elements often leads to serious pathophysiological conditions that may lead to growth impairment, reduced physical and mental power, and an increased risk of developing chronic diseases, to name only a few [7,8,9,10]. However, it has to be noted that the true metabolic demands remain unknown for most of the essential trace elements, and it was only for safety reasons that upper intake levels have been defined for most of the essential trace elements [11]. Upon metabolization, such elements are able to enter various biochemical pathways, and their interaction or conjugation with essential components like DNA or proteins can ultimately lead to structural and functional glitches [12]. Very common byproducts of these interactions represent free radicals like reactive oxygen species (ROS), which can subsequently damage a variety of cellular and molecular components in a wide range of pathways and are ultimately also responsible for the development of cancerous lesions, neurological disorders, or endocrine abnormalities, to name only a few [13,14,15,16]. However, mechanistically the generation of such radicals varies for each type of metal and, therefore, the radical species that are generated also depend on the respective metal involved [17,18,19]. Potential toxic trace elements can be classified according to their importance for physiological and biochemical processes as either essential (e.g., selenium (Se), zinc (Zn), copper (Cu), manganese (Mn), chromium (Cr), iron (Fe), molybdenum (Mo), and nickel (Ni)) or non-essential/toxic (e.g., aluminum (Al), cadmium (Cd), and lead (Pb)). The toxicity of trace elements are dose-dependent, and while non-essential elements may cause harmful effects at minute concentrations, essential elements exhibit toxicity only at higher concentrations not usually reached under physiological conditions.

The first indications that trace elements could play a role in the development of cardiovascular diseases already existed at the end of the 1960s [20]. Statistical analyses in selected countries like Japan, the UK, Sweden, Ireland, and others revealed correlations between the hardness of drinking water and the incidence of certain cardiovascular diseases [21,22,23]. This observation was later confirmed when striking differences were found in the mineral content of infarct tissue of the heart [24]. Today, the pathologies of the cardiovascular system are considered to be diseases of global relevance. In the addition, several epidemiological studies have also been published recently that deal with the topic of heavy metal trace elements in connection with the development of various diseases or, more specifically, cardiovascular disorders [25,26,27].

The pathophysiology of different heavy metal toxidromes is relatively similar. In most cases, heavy metals bind to oxygen, nitrogen, and sulfhydryl groups in various proteins, ultimately leading to alterations in enzymatic activity. Some metals also generate free radicals that can damage and degrade important cellular proteins, membranes, and organelles. The result of these various mechanisms is damage to various organ systems, such as the kidneys, nervous, respiratory, and cardiovascular systems [28,29,30,31]. In addition to directly interfering with the metabolic pathways of cells, the direct introduction of epigenetic changes upon trace element exposure by provoking both hyper- and hypo-methylation events has also been described [32,33,34]. In the case of Ni, the introduction of changes has been attributed to the direct inhibition of DNA methyltransferase enzymes [35]. Furthermore, new findings have been presented recently that show how exposure to metals can also cause epitranscriptomic dysregulation. These represent a possibly previously unrecognized new mechanism that could additionally be responsible for metal toxicity and carcinogenesis [36].

The prognosis and treatment of such diseases are often very lengthy and treatment-intensive for patients. Despite the availability of medical therapies and surgical measures, these diseases are very often fatal and therefore represent a major burden for the healthcare systems of many countries. This is just one reason why medical research in this field is of such great importance. This review therefore provides an overview of the latest developments, mainly in the last decade, in relation to the physiological functions and pathophysiological aspects of selected trace elements in connection with the development of cardiovascular diseases.

## 2. Essential Trace Elements

Trace elements are of great importance for a multitude of cellular functions at the biological, chemical, and molecular levels. They make essential biochemical reactions possible by acting as cofactors for enzymes and by serving to stabilize the three-dimensional structures of enzymes and proteins. Trace elements, however, have a dual importance. In normal concentrations, they are indispensable for proper physiological functions. However, disturbed concentrations can stimulate alternative metabolic pathways and even cause disease [37,38].

### 2.1. Selenium (Se)

Se is an essential trace element in the body. The Se-containing protein glutathione peroxidase exhibits central roles in regulating the physiological antioxidant status and plays additional roles in the thyroid metabolism and regulation of the immune response [39]. Reduced glutathione peroxidase activity could be related to the generation of toxic lipid peroxides, leading to endothelial dysfunction and arterial stiffness [40,41,42].

### 2.2. Zinc (Zn)

Zn is an essential trace element and its main sources in food are, e.g., meat, milk, eggs, and fish. Zn is required for a multitude of physiological processes, which is reflected by the fact that there are more than 300 enzymes that require Zn for their catalytic action. It plays a central role in nucleic acid and protein synthesis as well as regulating the transcription of genes as a co-factor for Zn-sensitive transcription factors [43]. It therefore represents an essential trace element that exerts essential roles in physiological and biochemical processes [44]. The increased intracellular accumulation of Ca^2+^ ions leads to stiffened arteries, and the respective deficiency could reduce vasodilatation at the receptor level [45,46,47].

### 2.3. Copper (Cu)

Cu is, after Fe und Zn, the third most abundant essential trace element in the human body. It is also widely distributed in nature and usually found in the form of minerals, rarely in a more native state. Cu exists in two oxidation states as Cu(I) and Cu(II). Its ability to gain or lose an electron characterizes its role in energy transfer processes in, e.g., cellular respiratory chain reactions. In addition, Cu functions as a cofactor for many enzymes and is involved in neurotransmitter synthesis and energy metabolism, as well as the cross-linking of extracellular matrix proteins like collagen and elastin [48]. Due to its role as an essential component of Cu-metalloenzymes, multiple functions in the hematologic, vascular, antioxidant and neurologic pathologies have been described. These include, e.g., increasing the cholesterol content of blood serum and disturbing the crosslinking of elastin fibers in blood vessels [49,50].

### 2.4. Manganese (Mn)

Mn functions as a cofactor of many enzymes, e.g., hydrolases, ligases, and lyases, and is central in metabolic processes such as protein glycosylation and lipid synthesis. Mn is a cofactor of the enzyme superoxide dismutase and therefore plays a central role in the detoxification of free radicals [51]. There are several reports that Mn exerts its vasculoprotective activities via a reduction in oxidative stress [33,34].

### 2.5. Chromium (Cr)

Cr occurs in multiple oxidation states [52]. On the one hand, Cr(VI) is mostly associated with different pathologies; on the other hand, Cr(III) is necessary during the metabolism of lipids and proteins and also acts as a cofactor for the action of insulin [53,54]. Cr can cause a multitude of pathologies through bioaccumulation in tissues and organs [55]. The association between Cr(VI) toxicity and lung cancer in steel industry workers is well established [56].

### 2.6. Iron (Fe)

Fe is the most abundant trace element in the human organism and almost 70% of the body’s total Fe is contained in heme proteins such as hemo- and myoglobin. Besides playing a central role in the transport of oxygen, Fe is also involved in numerous physiological processes such as mitochondrial respiration and oxidative phosphorylation and acts as an essential cofactor of many enzymes and functional proteins, e.g., cytochrome C oxidase, cytochrome P-450, and catalase [57]. Inappropriate Fe overload or deficiency correlates to a wide range of cardiovascular diseases (CVDs). Fe deficiency can impair cardiomyocyte mitochondrial function and energy supplement, leading to cardiac dysfunction [58,59].

### 2.7. Molybdenum (Mo)

The element Mo is complexed by a special cofactor to gain catalytic activity and is therefore an essential part of several enzymes (e.g., sulphite oxidase, xanthine dehydrogenase, aldehyde oxidase) [60]. High uric acid levels correlating with a high serum Mo activity are described as a marker of inflammation and endothelial dysfunction [61,62].

### 2.8. Nickel (Ni)

Traces of Ni are proven to be essential, at least for animal physiology, exerting a wide range of effects, including growth, senescence and Fe-uptake. However, a deficiency state in humans has not yet been clearly defined [63,64]. Ni as an immunotoxic and carcinogenic agent can cause a variety of health effects, such as contact dermatitis, cardiovascular disease, asthma, lung fibrosis and respiratory tract cancer [65].

## 3. Non-Essential/Toxic Trace Elements

### 3.1. Cadmium (Cd)

Cd is a toxic non-essential transition metal that poses severe health risks for both humans and animals and exerts no physiological functions [66]. It is present naturally in the environment and its concentration is increased in areas with industrial activities. The main use of Cd today is in batteries, but it is also an essential component in paints and a stabilizing agent in various plastic parts. One of the major source for increased Cd uptake occurs through the smoking of tobacco products [67,68]. For nonsmokers, Cd-rich food is the main source of uptake [69]. After uptake, Cd is distributed throughout the body and accumulates in different organs to varying degrees. Egger et al., showed the average concentration of Cd in the human body in a variety of organs and revealed new Cd pools and identified adipose and muscle tissue, although accumulating lower concentrations, as important pools due to their mass. This study was conducted on four fresh, non-embalmed human bodies (two males and two females) with a maximum post-mortem time of 48 h. Measured Cd concentrations in samples (μg kg^−1^) of the four donors in tissues of the cardiovascular system were as follows: heart muscle, 35; artery (radial), 63; aorta (ascendens), 94; and aorta (abdominal), 200 [70]. Cd is considered a highly toxic metal, and Cd and its compounds are classified by the International Agency for Research on Cancer (IARC) as a Group 1 carcinogen [71]. In recent decades, elevated Cd blood levels have been linked to various diseases in the human body, including pathological changes in the cardiovascular system, for example, in the development of atherosclerosis [72,73] and heart fibrosis [74]. In 2001, Abu-Hayyeh et al., showed that the aortic wall of smokers was, until that point, an underestimated target for Cd accumulation in the human body. In this study, total cadmium content was associated with smoking, assessed as pack-years, but was similar in aneurysmal and undilated aortas. However, a strong correlation between medial Cd content and the pack-years of smoking was detected. In vitro assays using aortic smooth muscle cells cultured on fibrillar collagen demonstrated that Cd was able to inhibit DNA synthesis and collagen synthesis and diminish cell numbers (IC(50) 2 μmol/L, 6 μmol/L, and 6 μmol/L, respectively), but higher concentrations of cadmium were required for the upregulation of metallothionein (EC(50) 23 μmol/L) [75]. The majority of negative effects on the cardiovascular system are based on the cytotoxic effects of Cd. Bernhard et al., showed in 2006 that the exposure of human primary arterial endothelial cells leads to a change in the cellular gene expression influencing cell shape and immune defense. In this study, 56 individuals were grouped into four subgroups based on their smoking habits. The data from this study suggest that increased levels of Cd are the result of the direct delivery of Cd to the human body by cigarette smoke and the Cd accumulates in the vasculature and changes arterial endothelial cell transcription [68]. Since that time, further papers have been published which substantiate that Cd is a major contributor to the development of atherosclerosis. Atherosclerosis, in turn, is involved in the development of various vascular diseases. Through a human study, an animal study and in vitro experiments with endothelial cells, Messner et al., were able to identify Cd as a novel and independent risk factor for the development of atherosclerosis. In the 195 young, healthy women of the Atherosclerosis Risk Factors in Female Youngsters (ARFY) study, Cd level was independently associated with early atherosclerotic vessel wall thickening. Similarly, Cd-fed ApoE knockout mice yielded a significantly increased aortic plaque surface compared to controls (9.5 versus 26.0 mm (^2^), *p* < 0.004). In vitro results indicated that physiological doses of Cd increased vascular endothelial permeability up to six-fold via (1) the inhibition of endothelial cell proliferation and (2) the induction of a caspase-independent but Bcl-xL-inhibitable form of cell death more than 72 h after Cd addition. Both phenomena have been shown to be preceded by Cd-induced DNA strand breaks and a cellular DNA damage response. The pro-atherosclerotic effect of Cd was therefore shown to be due to the inhibition of cell proliferation as well as the induction of DNA damage-induced cell death [72]. A study published in 2011 showed that Cd also promotes vascular inflammation, thus contributing to the progression of atherosclerosis. In this study, an in-depth histological analysis using light and scanning electron microscopy was performed on 18 sections taken from six cadmium-fed ApoE^−/−^ mice and 12 sections from five littermates not exposed to Cd. The Cd-fed mice showed a marked increase in lesion load (plaque area) and severity, and several inflammatory markers studied (CD68, CD3, CD25, vascular cell adhesion molecule 1 (VCAM-1), and heat shock protein 60 (Hsp60)) yielded a higher expression in Cd-fed mice. Statistical differences were identified for VCAM-1 and Hsp60 (*p* = 0.03 and *p* = 0.02). [73]. A further study by Messner et al., indicated that the mode of Cd-induced cytotoxicity is much more complex than previously anticipated. In summary, the incubation of endothelial cells triggers complex signaling pathways involving autophagy and apoptosis signaling that ultimately culminates in cell necrosis [76,77]. As mentioned above, the development of vascular atherosclerosis is a prerequisite for different cardiovascular events. The influence of Cd and other trace elements on various cardiovascular diseases is discussed in more detail in Section 4. Some of these diseases, but not all of them, are associated with the development of atherosclerosis and the abovementioned processes.

### 3.2. Aluminum (Al)

Al is the third most abundant element in the Earth’s crust. It has been used for centuries in the form of clay, glass and alum, but its industrial use began only at the end of the late 19th century. Despite its wide occurrence, Al is not essential for any physiological processes characteristic for life [78]. Al is known to inhibit more than 300 biological relevant reactions involving kinases or phosphatases, mostly due to its ability to bind to phosphates [79,80]. It mediates the extra mitochondrial release of free oxygen radicals, resulting in Fe-induced lipid peroxidation and protein denaturation [81,82].

### 3.3. Lead (Pb)

Pb is ubiquitous in our environment. However, no physiologic role in biological systems has yet been described [83]. Pb directly interferes with selected enzymatic activities, leads to competitive inhibition of absorption of essential trace elements, deactivates sulfhydryl antioxidant pools, and leads to an increase in arterial stiffness. Pb represents an exemplary environmental pollutant that exerts high toxic effects on many tissues and organs of exposed organisms. Pb toxicity is exerted via molecular mimicry with cellular cations and the generation of ROS. Due to its ability to replace Zn and Ca in proteins, it has the ability to interfere with essential physiological processes [84]. The main effects of Pb exposure include neurological, respiratory, and cardiovascular disorders [85,86]. These are usually based on inflicting disturbances during immune modulation as well as oxidative and inflammatory mechanisms and are associated with a multitude of diseases [87,88,89]. Regarding the influence of Pb on cardiovascular disease, the study by Zeller et al., has to be mentioned, in which the authors were able to show that Pb is a novel and importantly independent risk factor for vascular intimal hyperplasia, a prerequisite for growing atherosclerosis [90]. In a study that investigated the relationship between lead exposure and cardiovascular disease, mortality was evaluated in a cohort of 15,036 adults. It was reported that the estimated risk of dying from cardiovascular disease in association with blood lead levels was 3.76, 8.11, and 14.77 per 1000 person-years for patients in low, moderate, and high blood level cohorts, respectively [91].

### 3.4. Arsenic (As)

Inorganic As is present in groundwater used for drinking in several countries all over the world, whereas organic As compounds are contaminants primarily found in fish, representing the most relevant source of human exposure [92]. In addition, energy production from fossil fuel as well as the smelting of non-ferrous metals are major industrial processes that ultimately lead to the contamination of the environment by As [93]. While the concentrations in rural areas range from <1 to 4 ng/m^3^, concentrations in cities can reach values of 200 ng/m^3^. Even higher values of >1000 ng/m^3^ have been reported in special industrial or mining areas [92].

### 3.5. Mercury (Hg)

Hg is a common heavy metal pollutant found in the natural environment in different forms [94]. Inorganic Hg can be converted to organic compounds such as methylmercury, the latter being a very stable compound that can easily accumulate in the food chain. Therefore, the primary form of exposure for humans is via food, with fish and shellfish being the main source of methylmercury exposure [95]. The mechanism by which mercury exhibits toxic effects on the cardiovascular system is not yet fully understood, but the mechanism is believed to ultimately lead to an increase in oxidative stress levels within the cardiac tissues [96].

## 4. The Role of Trace Elements in Selected Cardiovascular Diseases

### 4.1. Abdominal Aortic Aneurysm

Abdominal aortic aneurysm (AAA) represents a serious health problem, is diagnosed in approximately 1.4% of people aged between 50 and 84, and is found to be almost six times more common in men than in women [97]. The dominating cause for developing an AAA is atherosclerosis and—to a lesser extent—also chronic inflammation or trauma [98,99,100,101]. Starting with the formation of atherosclerotic plaques, a weakening of the inner membrane of the aortic vessel due to a decrease in the density of elastin and collagen fibers can be detected. This results in a vessel wall that is susceptible to stretch-forces exerted by blood pressure, finally leading to the development of an aneurysm. Factors involved throughout this process include the activity of matrix metalloproteinases and the appearance of fibroblast apoptosis and inflammatory processes in the aortic wall [102,103,104].

In a recent study, the content of trace elements in the wall of AAA in respect to a coexisting iliac artery aneurysm (IAA) was evaluated. In this work, an association with a lower content of Ni as well as a significantly higher content of Cd was demonstrated. The levels of remaining trace elements like Cu, Zn, Mn, Mg, and Ca were detected at similar concentrations [105]. The authors of this study linked the higher Cd concentrations to an increased exposure to tobacco smoke [68,106]. However, the variation of the Ni contents in this study were found not to be statistically relevant. This was most likely due to the small size of the study group, as claimed by the authors, since the content of trace elements was assessed in samples of AAA walls harvested intraoperatively in only 19 consecutive patients [105].

One study investigated the influence of dietary habits and smoking on the level of Se and Pb in blood, the aortic wall and parietal thrombus of patients with aortic abdominal aneurysms [107]. Forty-nine patients with AAA prior to surgical procedures aged 42–81 years and a control group of twenty-two healthy volunteers aged 31–72 years as well as seventeen aortic wall samples from deceased individuals were included in this study. The authors reported significantly (*p* < 0.008) reduced mean Se levels in serum of patients with AAA (60.37 ± 21.2 cm/L) compared to healthy volunteers (75.87 ± 22.4 cm/L). There was also a significant correlation between the serum content of Se and the parietal thrombus of the examined patients (r = 0.69, *p* < 0.0001). It was also shown that the Se concentration in the aortic walls was inversely correlated to the concentration of Pb. Significantly lower concentrations (*p* < 0.05) of Se (39.14 ± 37.1 cm/g) and significantly higher (*p* < 0.05) concentrations of Pb (202.69 ± 180.6 cm/g) were detected in samples from the aortic wall of smoking patients compared to non-smoking patients (77.56 ± 70.0 cm/g and 73.09 ± 49.8 cm/g, respectively). The conclusion from this study was that the measured Se serum levels are lower in patients with AAA than in healthy control individuals. In aortic wall samples, Se concentration was demonstrated to be inversely correlated with the Pb concentration. Based on previous data, it was concluded that dietary habits as well as smoking exhibit a considerable influence on the Se and the Pb status in patients with AAA [108,109].

Another study demonstrated that intraluminal thrombus thickness is not related to the concentrations of trace elements in the wall of infrarenal abdominal aortic aneurysms [110]. It was shown that concentrations of Mg, Zn, Mn, and Pb in the wall of AAA were significantly increased in respect to the intraluminal thrombus (ILT) samples. Only Cu concentration was lower in the AAA wall compared with the thrombus. Moreover, the concentration of Ca, Zn, Pb, Cu, and Mg have been shown increase with ILT thickness. The authors of the study concluded that the ILT participates in the progression of AAA via mechanisms independent of trace element supply to the wall of the aneurysm sack.

Several model systems have initially supported the notion that Cu might also play a role in the development of aortic aneurysms. One example is the Blotchy mouse, characterized by a defect of Cu metabolism that includes a reduced activity of the Cu containing enzyme lysyl oxidase that is essential for cross-linking elastin [111]. In addition, it has been demonstrated that Cu deficiency in pigs can lead to spontaneous arterial rupture [112]. Furthermore, the Menkes’ syndrome is characterized by a reduction in elastic fibers in arterial walls as well as an abnormal Cu metabolism [113].

A study that addressed the question whether such a Cu deficiency was indeed associated with aortic aneurysms analyzed the respective Cu levels in liver and aortic wall samples from patients with aortic aneurysms. However, in this study, the concentrations of Cu were not different between the patients and the control group and an association with human aortic aneurysms could therefore not be supported [114].

### 4.2. Thoracic Aortic Dissection

Aortic dissection (AD) represents a life-threatening condition that is caused when the intimal layer of the aorta is ruptured. This results in the separation or dissection of the individual layers of the aortic wall and blood in the vessel spills into the media [115]. After the formation of a dissection, aortic inflammation enhances the dilation and subsequent rupture of the aorta due to the infiltration of inflammatory cells into the adventitia of the aortic wall and the concomitant degradation of ECM structures [116]. Due to the dramatic nature of this event, disease progress is rapid and the fatality rate is very high, reaching almost 50% within 24 h after the initial dissection and increasing with time [117,118].

There are several studies available indicating an association between Zn serum concentrations and the occurrence and respective outcome of cardiovascular diseases [119,120,121]. For example, it has been demonstrated that the Zn content of aortic wall tissue in patients with thoracic aortic aneurysm (TAA) was lower than in healthy individuals. In this study, a total of 108 patients (47 with abdominal aortic aneurysm (AAA), 61 patients with thoracic aortic aneurysm (TAA), and a control group of 20 abdominal aortic (AA) and 20 thoracic aortic (TA) wall samples from deceased patients) were studied. It was demonstrated that the mean concentration of Zn in the aortic wall of patients with TAA and AAA (12.9 ± 4.05 μg/g and 18.54 ± 12.3 μg/g, respectively) was significantly lower (*p* < 0.05) than in the control group samples [122]. This was confirmed by other studies demonstrating Zn deficiencies in serum as well as aortic tissues from patients with the corresponding aneurysms [123,124].

Zn exhibits a multitude of physiological effects, e.g., the induction of lymphopenia and compromised immune responses, caused by the presence of only low levels of this trace element [125,126]. A recent study aimed to the characterize the influence of Zn on the progression of thoracic aortic dissection through the process of the inhibition of inflammation [127]. The motivation for this study were observations that an inflammatory response is involved in the formation of the TAD and patients with serious symptoms exhibited higher activities of inflammatory cells in the aortic tissue compared to healthy individuals [128]. The hypothesis was that the accumulation of activated inflammatory cells within the vascular wall usually induces aortic weakening through the degeneration of extracellular matrix material. If Zn deficiency reduced tissue inflammation, an amelioration of AD should be observable. To achieve this goal, a β-aminopropionitrile monofumarate (BAPN)-induced TAD model was used to determine the effects of low Zn levels on the dissection process. And indeed, low Zn treatment was shown to attenuate the progression of TAD by improving AD formation and rupture, thereby reducing mortality. This was due to a down-regulation of aortic inflammation by attenuating the infiltration of macrophages, suppressing the switch of VSMCs from contractile to synthetic phenotypes, and eventually inhibiting TAD development.

A recent study deals with the finding that low levels of circulating and aortic tissue Fe are associated with vascular smooth muscle cells (VSMC) dysfunction and aortic instability [129]. Fe deficiency was reported to increase the incidence and severity of aortic dissection (AD) as well as dysregulation of VSMCs, most likely mediated through the integrin pathways. Moreover, it was also presented that congenital Fe deficiency was causative of vascular developmental disorders. Regarding Cd, no increased concentration was found in the tissues of aortic dissection patients [124].

### 4.3. Aortic Valve Sclerosis/Stenosis

Aortic valve sclerosis (AVS) is described as the calcification and concomitant thickening of aortic valve cusps without the obstruction of the ventricular outflow [130,131]. A recent study investigated the relationship between the development of AVS and the levels of selected trace elements like Fe, Zn, Se, and Cu. The authors were able to demonstrate a significant difference in the prevalence of diabetes and blood pressure levels as well as the body mass index between patients and healthy controls [132]. In this study, it was also shown that serum Zn concentrations in AVS patients are significantly reduced compared to those in the healthy control group, a finding in line with other studies that demonstrated lower Zn concentrations in patients with coronary artery disease, sclerotic heart valves, rheumatic heart disease, and heart failure [133,134,135].

Aortic valve stenosis (AS), on the other hand, represents the most common valvulopathy among adults [136]. It is characterized by inflammation, the remodeling of the extracellular matrix, and subsequent calcification that leads to a narrowing of the valve and the consequential obstruction of the cardiac outflow [137]. A recent study aiming to characterize valvular Fe in relation to pathological changes associated with AS and the effects on valvular interstitial cells (VIC) in terms of Fe uptake and Fe-induced responses [138]. Based on the findings of this study, the authors suggested that VIC and smooth muscle cells share the characteristics of an inducible Fe storage under pathological conditions, also linking Fe accumulation with increased VIC proliferation as well as promoting Wnt/β-catenin signaling in other cell types. This has been confirmed by a study demonstrating that Fe loading had an effect on Wnt signaling using the mutant adenomatous polyposis coli (APC) gene cell lines Caco-2 and SW480. In contrast, wild-type APC and beta-catenin-containing lines, HEK 293, and human primary fibroblasts were not responsive to iron loading. The verification of this finding was possible with SW480 cells that no longer induced iron-mediated Wnt signaling when transfected with wild-type APC. In addition, using the cell line LS174T, wild-type APC but mutant beta-catenin also proved responsive, suggesting that the role of Fe is to regulate beta-catenin. The authors therefore speculated that excess Fe could exacerbate tumorigenesis against the background of APC loss, a situation commonly observed in tumors [139,140,141].

In another study, the authors investigated the effects of Fe overload on the aorta of rats since excessive Fe has already been recognized as a risk factor for tissue damage. In one study, twenty 8-week-old male Sprague Dawley rats were randomly divided into two groups: (1) the Fe-overload group (n = 10), which received an intraperitoneal injection of Fe-Dextran (250 mg/kg of body weight) 5 days a week for 4 weeks, and (2) a control group (n = 10) that received an intraperitoneal injection of the same dose of 0.9% NaCl solution. Upon analysis, it was demonstrated that iron levels in serum (88.165 ± 15.830 μmol/L) and aortic tissue (10.494 ± 3.636 μmol/g protein) were higher in the iron-overload group than in the control group (17.338 ± 2.289, 6.507 ± 1.259) (*p* < 0.05) [142,143]. The authors demonstrated that these excessive Fe levels also led to subsequent renal and hepatic damage. In line with other published data, Fe metabolism-related factors were significantly changed during Fe overload, also leading to Ca deposition in the aorta, implying a key role in the pathophysiological process of vascular calcification by inducing osteoblast differentiation factors and downregulating the inhibitory factor for calcification [144].

The goal of another recent study focusing on trace elements in calcified valves in patients with acquired severe aortic valve stenosis was to determine the concentration of no fewer than 21 metals and trace elements [145]. Authors have been able to identify significantly higher concentrations of Ba, Ca, Cr, Mg, P, Pb, Se, Sn, Sr, and Zn as well as lower concentrations of Cd, Cu, Mo, S, and V in calcified aortic valves than in healthy controls. In addition, positive correlations of pairs of trace elements (Ca-P, Cu-S, and Se-S) as well as negative correlations (Mg-Se, P-S, and Ca-S) were demonstrated in affected valves structures. The authors concluded that some exposures might very well increase their accumulation and environmental burden over time, and the actual aortic valve calcification process should not be ruled out.

Interestingly, calcified aortic valves—as a prerequisite for aortic valve stenosis—contained lower amounts of Cd compared to healthy aortic valves (age-unadjusted model). Yet, analyzing sex differences revealed that female patients with aortic valve calcification had significantly higher Cd levels. However, it has to be kept in mind that the female patients in this study were significantly older than the male group [145].

Another mechanism through which Hg exerts toxic effects on the cardiovascular system is through the inactivation of paraoxonase, an extracellular antioxidative enzyme related to high-density lipoprotein (HDL). [146,147] This enzyme also plays an important role as an antioxidant of LDL, which directly links this process to the development of atherosclerosis and the increased risk of acute myocardial infarction, cardiovascular disease, coronary heart disease, and carotid artery stenosis. [148]

### 4.4. Heart Failure

Damage to myocardial tissue occurs mainly due to a lack of oxygen supply in the course of a myocardial infarction. However, heart failure is not only triggered by oxygen deprivation as a vast number of other harmful substances can also trigger the death of heart muscle cells. Cardiotoxicity characterized by the metal-induced death of cardiomyocytes has gained more attention in recent years [149]. This is also due to the fact that the cardiovascular system, and thus the heart, has long not been considered a significant target of metal ion toxicity. This view changed after the study by Egger et al., which showed that muscle tissue, including the heart muscle, was an (at the time) underestimated target for Cd deposition [70]. Recently, Ćirović et al., showed that patients with secondary cardiomyopathy had significantly higher concentrations of Pb, Ni, Mn, and Cu in their heart tissue than the group without cardiomyopathy. However, as the authors stated in their publication, cardiomyopathy is a complex diseases and this status cannot solely be explained by the presence and toxicity of the mentioned elements [150].

Pb, whose increased concentration in the left ventricle was noted by Ćirović et al. [150], was identified as severely cardiotoxic in an animal study [151]. In detail, Klinova et al., observed higher levels of angiotensin-converting enzyme (ACE) and an amplified T-wave amplitude during an electrocardiograph examination. In addition, the cardiomyocyte thickness of the lead-exposed group (5.38 ± 0.12 μm) was found to be higher than that of the control group (4.74 ± 0.08 μm); although this difference was relatively small, it was still statistically significant (*p* < 0.01) [151]. Moreover, they recorded a reduction in the maximal velocity of thin filament slide over myosin, leading to a reduced contraction of the cardiomyocytes. In vitro, the exposure of cardiomyocyte to Pb-induced toxicity through the induction of apoptosis [152]. In 2013, Turdi et al., also observed abnormalities in the contractile function of cardiomyocytes after Cd treatment [153].

Cu, known for its actually beneficial effect at lower concentrations, exerts toxic effects at increased concentrations and cardiac-specific accumulation. Pan et al., were able to show that the long-term exposure of mice with Cu resulted in the mitochondria-mediated apoptosis of cardiomyocytes and, therefore, heart damage. In detail, the authors were able to characterize a negative effect of Cu on the extracellular matrix within the heart as well as damage to the mitochondrial membrane, leading to the induction of cardiomyocyte death [154]. Toxic effects have also been reported with increased exposure to Mn and Ni, with the latter suspected being responsible for congenital heart defects in offspring [155,156].

The vast majority of data linking metals and cardiotoxicity are available for Cd, with the number of publications having increased significantly, especially in the last 10 years. Below we quote some of the most important of these studies, although we cannot claim that this list is exhaustive. In 2015, Borné et al., published a population based prospective cohort study consisting of 4378 participants without a history of heart failure or atrial fibrillation (aged 46–67 years, 60% women) showing that elevated blood Cd levels are associated with an increased incidence of heart failure [157]. In 2015, our own group was able to show that high cholesterol levels together with elevated Cd exposure is a risk factor for significant heart fibrosis in ApoE knockout mice, mainly due to cardiomyocyte cell death following scar tissue formation. For in vitro and in vivo experiments, the HL-1 cardiomyocyte cell line as well as female C57BL/6J mice and female ApoE^−/−^ mice were used. [74]. Similar effects have been demonstrated by Ghosh et al., in albino Wistar rats. The intragastric administration of Cd-induced inflammatory responses, apoptosis, and distorted myofibril arrangement as well as the vacuolization and congestion of vessels within cardiac tissue. Amongst others, these processes include the induction of oxidative DNA damage, damage to proteins and lipids, and decreased overall antioxidant activity [158]. In addition, in vitro studies using cardiomyocyte culture (with either primary cells or cell lines) reached similar conclusions. Chen et al., demonstrated that the incubation of cardiomyocytes with Cd resulted in ER stress and negatively influenced the energy homoeostasis of the cells [74,159].

Oluranti et al., also demonstrated an adverse effect of Cd on cardiomyocyte metabolism in a rat study. In detail, Cd leads to a reduction in fasting serum insulin levels and the reduced activity of pyruvate and hexokinase, while the activity of pyruvate dehydrogenase significantly increased after treatment [160].

As mentioned previously, the administration of Cd to rats, in addition to its effect on cardiomyocyte physiology, also leads to significant and pathological changes in the extracellular matrix of cardiac tissue. Das et al., in 2021, examined a Cd-induced imbalance in the MMP-TIMP system, potentially induced by inflammatory signals. Consequently, the authors hypothesized that this processes potentially contributes to various cardiovascular pathologies [161]. Chou et al., describing the detailed analyses of cardiac tissue that revealed inter alia focal necrosis, perivascular and interstitial fibrosis, and irregular sarcomere structures, recently found similar effects. As reported by many other studies, they observed the apoptosis of cardiomyocytes and increased expression of MMPs (in this case MMP-14). The authors of this study then extended their analysis to the aortic tissue and included in vitro cell experiments. Through this, the authors demonstrated damage to the intima and media of the aorta, potentially induced by the reduced viability of smooth muscle cells, as shown by in vitro experiments [162].

Interestingly, other metals can also neutralize the effect of Cd. For example, Feng et al., demonstrated that Se is able to protect against Cd-induced cardiac injury by disturbing the cell death signal pathway induced by Cd. In this study, 40 rabbits were randomly divided into four groups: the control group, the Se (0.5 mg kg^−1^·body weight (BW)) group, the Cd (1 mg kg^−1^·BW) group, and the Se + Cd group. After 30 days of feeding, morphological changes, the levels of oxidative stress and myocardial enzymes, the content of cardiac troponin T, programmed cell death (pyroptosis, autophagy, and apoptosis), and PI3K/AKT/PTEN transduction capacity were observed. These study results showed that Cd impaired the physiological balance of trace elements and caused myocardial damage, increased cardiac oxidative damage, and led to programmed cell death. The co-administration of Se prominently ameliorated histological lesions and improved the cardiac function of hearts in Cd-induced rabbits [163]. Finally, the study by Fitch et al., is worth mentioning, as they exposed female and male C57Bl/6J mice to Cd (5 mg/L ad libitum) and analyzed cardiac function after 8 weeks of treatment. The results of this study were surprising, as they showed that male mice featured a reduced left ventricular ejection fraction and a fractional shortening after treatment, together with an increased ventricular volume at end-systole and a decreased inter-ventricular septal thickness at end-systole. Mechanistically, the authors were able to show an influence of Cd on the ER, i.e., the decreased protein expression levels of sarco/endoplasmatic reticulum Ca^2+^-ATPase 2a (SERCA2a). The surprising results, however, were not the Cd-induced changes in the heart but that these occurred only in the male mice and not in the female mice [164].

As can be seen from this section on Cd and its negative effects on cardiac muscle tissue, numerous studies have already been published that demonstrate both the effects and the mechanisms behind it. Nevertheless, further studies in this area are absolutely necessary, as not all effects and their consequences are yet fully understood, as shown by the recent study by Fitch et al. [164].

## 5. Conclusions

Cardiovascular disease is the leading cause of death worldwide, and an increasing number of studies point to the central role that trace elements play in the pathophysiology of symptom development and progression. However, much more work is needed to better understand the pathophysiological processes down to the molecular details. An additional important topic that will become even more relevant in the near future in connection with legalization efforts concerns the chapter on the consumption of marijuana or cannabis. The use of these substances is on the rise worldwide, which is significant in that the marijuana plant is known to accumulate Cd and Pb from the soil in very high quantities. A recent article addressed this issue and reported on general correlations between internal metal levels and exclusive marijuana use [165]. This makes the issue of exposure to such metals all the more relevant in the context of cardiovascular disease and is likely to become increasingly important to human health in the coming decades. It also remains critical to further elucidate and describe the specific mechanisms by which trace elements interfere with normal physiological processes, especially in light of recent findings that trace elements are able to induce changes at the epigenomic and the epitranscriptomic levels. This underlines the importance of the omnipresent environmental pollution caused by nanoparticles and microplastics in combination with trace elements, which has become even more significant in recent years. Such particles dispersed in the environment have long been recognized as an environmental problem. However, the various effects of the interaction between plastics and the environment remain difficult to reconstruct and further research in this area is urgently needed. This could ultimately help to correctly classify the specific hazards of the role of trace elements in environmental pollution. In addition, the mechanisms derived from these findings could also help to identify further therapeutically relevant targets for future medical applications with regard to the prevention and treatment of cardiovascular diseases and other pathophysiological processes.
